# Selling visibility-boosts on dating apps: a problematic practice?

**DOI:** 10.1007/s10676-023-09704-y

**Published:** 2023-05-18

**Authors:** Bouke de Vries

**Affiliations:** 1 Department of Philosophy, University of Zurich, Zurich, Switzerland; 2 Department of Philosophy and Moral Sciences, Ghent University, Ghent, Belgium

**Keywords:** Dating apps, Visibility boosts, Tinder, Romantic relationships, Love, Gambling, Addiction, Autonomy, Socio-economic justice

## Abstract

Love, sex, and physical intimacy are some of the most desired goods in life and they are increasingly being sought on dating apps such as Tinder, Bumble, and Badoo. For those who want a leg up in the chase for other people’s attention, almost all of these apps now offer the option of paying a fee to boost one’s visibility for a certain amount of time, which may range from 30 min to a few hours. In this article, I argue that there are strong moral grounds and, in countries with laws against unconscionable contracts, legal ones for thinking that the sale of such visibility boosts should be regulated, if not banned altogether. To do so, I raise two objections against their unfettered sale, namely that it exploits the impaired autonomy of certain users and that it creates socio-economic injustices.

## Introduction

Love, sex, and physical intimacy are some of the most desired goods in life and they are increasingly being sought on dating apps such as Tinder, Bumble, and Badoo. For those who want a leg up in the chase for other people’s attention, almost all of these apps now offer the option of boosting one’s visibility for a certain amount of time, which may range from 30 min to a few hours. For example, Tinder, currently the largest dating app in the world with 66 million average monthly active users (Curry, 2021; Reuters, 2021), sells ‘Boosts’ that are said to allow you to ‘be one of the top profiles in your area for 30 min’ and to ‘get up to 10x more profile views while boosting’ (*Boost*, n.d.) as well as ‘Super Boosts’ that are said to allow you to ‘cut to the front and be seen by up to 100x more potential matches’ (*Super Boost*, n.d.). (A match being a situation where two dating-apps users have liked each other’s profile, which on some apps must exist before users can communicate on the platform.)

Such special treatment does not come cheap. Consider again Tinder’s Boosts and Super Boosts; although the precise costs of these visibility boost varies depending on one’s geographical location and age (with the exception of California were all age-groups now pay the same fees after an age-discrimination lawsuit was brought that Tinder settled for 17.3 million USD) (Vega, 2019), a 32-year-old person from The Netherlands (such as yours truly) pays 4 EUR for a single Boost of 30 min; 19 EUR for a bundle of five Boosts; and 30 EUR for a bundle of 10 Boosts. Should this person want greater visibility still, he or she will need to pay a hefty 30 EUR for a 3-hour Super-Boost; 53 EUR for a 6-hour one; and 98 EUR for a 12-hour one, which in the last two cases will well exceed the monthly subscription fee that users with these characteristics pay for the most expensive membership (30 EUR for a Tinder Platinum membership).

In this article, I argue that there are strong moral grounds and, in countries with laws against unconscionable contracts, legal ones for thinking that the sale of visibility boosts ought to be regulated, if not banned altogether. To do so, I raise two objections against their unfettered sale, namely that it exploits the impaired autonomy of certain users (Sect. 2) and that it creates socio-economic injustices (Sect. 3). The final section concludes (Sect. 4).

## The impaired autonomy-objection

While the freedom to make contracts is a great good on which much of our economic prosperity and welfare depends, few countries give legal persons full discretion over the terms of their contracts. In most jurisdictions, there are laws that seek to ensure a threshold level of fairness (cf. Thal, 1988). My aim in this section is to suggest that there are good grounds for thinking that the unfettered sale of visibility boosts falls short of this threshold by violating an equity doctrine known in common law jurisdictions as ‘unconscionability’. One of the clearest expressions of this doctrine was provided by Justice Mason in *Commercial Bank of Australia Ltd v Amadio* (1983, p. 461):

Relief on the ground of unconscionable conduct will be granted when unconscientious advantage is taken of an innocent party whose will is overborne so that it is not independent and voluntary, just as it will be granted when such advantage is taken of an innocent party who, though not deprived of an independent and voluntary will, is unable to make a worthwhile judgment as to what is in his best interest.

As Mason, following Justice Fullager, went on to note, there are various factors that can impair people’s ability to make such worthwhile judgements that may offer a ground for relief based on unconscionable conduct (*Commercial Bank of Australia Ltd v Amadio*, 1983, p. 462). As Fullagar had stated in *Blomley v Ryan* (1956, p. 405):

The circumstances adversely affecting a party, which may induce a court of equity either to refuse its aid or to set a transaction aside, are of great variety and can hardly be satisfactorily classified. Among them are poverty or need of any kind, sickness, age, sex, infirmity of body or mind, drunkenness, illiteracy or lack of education, lack of assistance or explanation where assistance or explanation is necessary. The common characteristic seems to be that they have the effect of placing one party at a serious disadvantage vis-a-vis the other.

My aim in what follows is to argue that the unfettered sale of visibility boosts on dating apps is likely to put a subset of buyers at such a disadvantage by exploiting one or more ways in which their autonomy is impaired, whereby ‘autonomy’ can be defined broadly as independently endorsing a conception of the good life and living in accordance with it (Colburn, 2010). These include dating app-users with proclivities for developing gambling addictions, as the use of visibility boosts – I want to suggest – is plausibly construed as a non-conventional type of gambling, which I understand to be ‘the act of wagering or betting money or something of value on an event with an uncertain outcome with the intent to win more money or things of value than was wagered’ (Winters et al., 2012, p. 18).^1^ For the purposes of this paper, is not necessary to defend a specific conception of ‘addiction’, the correct understanding of which is hotly debated within scholarly literature (e.g. Henden et al., 2013; Hogarth, 2020; Vandaele & Ahmed, 2021; Wiers & Verschure, 2021). All I assume here is that, contrary to the picture of addiction painted by the moral model on which addiction is presented as ‘a choice characterized by voluntary behavior under the control of the addict’ (Henden et al., 2013, p. 1), addictive behaviors are not rarely – but perhaps not invariably (I remain non-committal on this) – largely outside of the addict’s control, whether this is best explained by the habit model of addiction; the compulsion model; the brain disease model; or some other model still (cf. Hogarth, 2020; Vandaele & Ahmed, 2021; Volkow et al., 2016).

To show that the use of visibility boosts constitutes a (non-conventional) type of gambling that is likely to be addictive to a portion of buyers in such an autonomy-impairing sense – and whenever I speak of ‘addiction’ from hereon, I will be referring to autonomy-impairing forms of addiction – it is instructive to consider the similarities between this activity and a type of gambling whose addictive potential has been proven (Schüll, 2012), with some studies reporting it to be the single-most addictive type (for an overview, see Turner & Horbay, 2004, p. 13; but cf. Dowling et al., 2005): Playing slot machines.

- Whether it is spins on the machine or visibility boosts that are being bought, the buyer can win something that has more value to them than was wagered, which it was noted is a *sine qua non* of gambling, namely cash prizes and ‘likes’ from other dating app-users respectively.
- In both cases, there exists a variable reinforcement schedule, which is another essential component of gambling (see the previous paragraph) and part of what makes this activity addictive to some (M. Brooks, 2019). While this will be obvious with respect to slot machine games of which the outcome of any single game cannot be known by the players in advance even if they can be confident to lose money overall if they play the game frequently enough, it also applies to visibility boosts. To see this, it should be noted that, rather than guaranteeing a fixed number of additional likes, how many more likes, if any, individuals receive relative to a scenario where they do not boost is variable as e.g. Tinder openly acknowledges on its website.^2^
- In both cases, people will find out how much, if anything, they gained from their purchase. In the case of slot machine games, this is indicated by the number of coins that are paid out by the machine, whereas in the case of visibility boosts, it is indicated by the number of additional likes that users receive, which on Tinder are marked by a purple lightning bolt-icon to allow users to differentiate them from likes acquired without the aid of a boost (*Used Boost or Super Boost, and Didn’t Get Any New Matches*, n.d.).
- In both cases, there is a high ‘event frequency’ in that people quickly discover whether their gamble has paid off or is paying off, which has been found to make gambling more addictive (e.g. Harris et al., 2021; Linnet et al., 2010). Whereas slot machine games only take a few seconds to play, booster-aided likes can, and sometimes do, come in as soon as users activate their boost.

In response, it might be argued that there exists a relevant difference between the use of visibility boosts and the playing of slot machines that prevents the former from qualifying as a type of gambling and that may consequently lead some to doubt its addictive potential. Since dating app-users can choose their profile pictures and decide about the content of their accompanying biographies (subject to the dating app’s guidelines, of course, which normally prohibit things such as full nudity and hate speech), they have the opportunity to present themselves in ways that will increase their chances of amassing (additional) likes. From this, a critic may conclude that, unlike slot machine games whose outcomes players cannot influence, the use of visibility boosts is better thought of as a *game of skill* rather than a game of chance.

One problem with this reply is that even if dating app-users are able to exercise *some* control over the success of their boosts, the exact number of additional likes that they receive on each occasion, if they receive any at all, will still vary as we saw Tinder acknowledges on its website, which is borne out by the experiences of users (*[Guys] How Many Matches Do You Get from a Boost?*, 2018). What this means is that *no matter how strong* people make their profiles, visibility boosts will continue to follow the logic of variable reinforcement in that for any given state of someone’s profile, the outcome of boosting at t1 need not, and often will not, be the same as the outcome at t2 (cf. M. Brooks, 2019). But that is not all. The abovementioned argument also goes wrong in assuming that having a certain degree of control over the outcome of a game *suffices* to render the game non-addictive, which is clearly false. Consider poker; despite involving a fair amount of skill, this game is still classified as a game of chance in many countries and has been shown to be addictive to some players (e.g. Griffiths et al., 2010).

Another way in which the analogy with slot machines might be challenged is to say that visibility boosts do not allow the user to win money or anything that can be exchanged for money, such as casino chips, and that, because of this, they are unlikely to be addictive to anyone. Perhaps the best way to debunk this argument is to point out that loot boxes in videogames such as Overwatch, Counterstrike and FIFA – which are ‘digital containers of randomised virtual items’ that are usually bought for real money and that help individuals to play the game in question, for example by providing them with virtual weapons, armor, or high-quality football players (Drummond et al., 2020, p.986) – do not allow people to win money or anything that is convertible to money either. Still, there is an increasing number of studies showing that they are ‘psychologically and structurally akin’ to conventional forms of gambling (Close & Lloyd, 2021, p. 37; cf. Drummond & Sauer, 2018) and, as one would expect in light of this, to be highly addictive for a subset of players (Brady & Prentice, 2021; Brooks & Clark, 2019; Drummond & Sauer, 2018; Zendle & Cairns, 2018, 2019), which led Belgium recently to recognize their consumption as a form of gambling.^3^

At this point, a critic might concede that there is strong reason to suspect that visibility boosts can be addictive to (some) users but still deny that this renders their unfettered sale unconscionable. To support this view, said critic might argue, first, that as (normally-functioning) adults, we are capable of autonomously deciding whether to sign up to a dating app and, second, that having this ability is *enough* to hold us responsible for any addictions that we may subsequently develop to these apps and to the visibility boosts sold on many of them in particular. A real-life example of this type of reasoning can be found in *Kakavas v Crown Melbourne Ltd* (2013). In this case, the Australian High Court dismissed claims by the plaintiff, Harry Kakavas, who had a long history of gambling problems and made losses at the Crown Casino in excess of 20 million AUD, that the casino had acted unconscionably by incentivizing him to gamble by offering him, among other things, special rebates, a line of credit, and the use of a private jet. Although Kakavas’ history of gambling problems was known to Crown, having previously denied him access to its venue during the 1990s, the Court held that:

[W]e do not accept that the appellant’s pathological interest in gambling was a special disadvantage which made him susceptible to exploitation by Crown. He was able to make rational decisions to refrain from gambling altogether had he chosen to do so. He was certainly able to choose to refrain from gambling with Crown (*Kakavas v Crown Melbourne Ltd*, 2013, p. 135).

What to make of the critic’s view? Whether the responsibility-to-self-exclude argument is convincing when it comes to gambling in casinos, I believe that it is unconvincing as far as the sale of visibility boosts on dating apps is concerned despite the superficial similarities between the two practices. To see why, it should be observed that, unlike the activity of gambling or any success therein, the intimate relationships that a large portion of us seek on dating apps have a profound impact on our wellbeing and health (Ge et al., 2020) and, as such, represent *fundamental human needs* that according to Maslow’s famous hierarchy of needs are even more important than our interests in esteem and self-actualisation (Maslow, 1943). When we take this into account along with the fact that a substantial share of early stage-dating happens on these apps nowadays – in the United States, for instance, almost half of young adults aged 18–29 reports having used a dating app (Vogels, 2020), whereas in the United Kingdom, it was found that even before the COVID-19 pandemic (which saw an accelerated rise in the use of online dating services; Meisenzahl, 2020), more relationships among 18–35 year olds were initiated online (23%) than were initiated at work (20%); via a mutual friend (19%); or at a bar, pub, or club (17%) – it becomes clear that eschewing the services of dating apps – but not those of casinos – carries high costs for many people.

Now, all this would be immaterial were it not for the fact that almost all the largest dating apps, including Tinder, Bumble, OK Cupid, Grindr, and Badoo, have started to sell visibility boosts, often employing intrusive marketing tactics to do so (for example, Tinder sends regular notifications to users urging them to boost). Consider the online dating market in The Netherlands, where 62% of dating app-users report to be on Tinder followed by Badoo at 23%, Happn at 18%, and Lexa at 13% (Statista, 2019). Since all these apps are selling visibility boosts, residents of this country who exclusively wish to use dating apps that do not, such as Happy Pancake, would need to use apps that are used by less than 13% of the online dating community, which is a small percentage indeed for those who are serious about finding love online. In short, even if we can be reasonably expected to stay away from casinos to avoid developing a potential gambling addiction, it is doubtful whether the same is true of dating apps that sell visibility boosts, given that, for many of us, these apps are the main (initial) gateway to fulfilling our fundamental interests in intimate relationships. (In this regard, a parallel might be drawn with the sale of alcohol within supermarkets. Part of the reason why e.g. New Zealand has introduced regulations forbidding supermarkets from selling alcohol within prominent areas, such as check-out counters (see its Sale and Supply of Alcohol Act of 2012), and why this country’s medical authority wants to ban the sale of alcohol within these stores altogether (Roy & Jong, 2017) is that supermarkets are costly to avoid for many of us due to their role in helping us to fulfil our fundamental interests in adequate nutrition.)

Having focused hitherto on how gambling addictions might prevent people from deciding autonomously about whether to buy visibility boosts, I should stress that such addiction is *not the only possible autonomy-inhibiting factor* that might influence these purchases. Research has shown that loneliness coupled with a preference for online communication is conducive to compulsive dating app-use (Coduto et al., 2020). Since visibility boosts increase people’s chances of receiving (more) likes and are frequently marketed aggressively to them (see the previous paragraph), this raises the worry that some dating app-users lack autonomous control over their purchases of such boosts by virtue of being desperate for social connection. Likewise, the fact that a large share of individuals report using dating apps to be validated by others (Alexopoulos et al., 2020; Timmermans & De Caluwé, 2017) – for example, one young woman describes how her ‘sociopathic curiosity and appetite for constant validation is fuelled by Tinder’s addictive function,’ which has caused her to start ‘consuming hundreds of profiles on boring journeys or in queues for a slow barista’ (quoted in Kent, 2015) – raises concerns that some of those with pathological needs for validation are buying visibility boosts in ways that fail to reflect autonomous agency.

Before turning to another objection to the unfettered sale of visibility boosts, a potential rebuttal to the current one must be addressed. This rebuttal is premised on the supposition that dating app-companies *cannot know* which of their users are unable to decide autonomously about whether to buy visibility boosts. To the extent that this is so and such companies have legitimate interests in selling visibility boosts to non-addicted users, it might be concluded that *even if* some users are unable to decide autonomously whether to buy them, the unfettered sale of such boosts cannot be unconscionable.

I find this argument unconvincing. For one thing, the fact that dating app-companies gather large amounts of user-data (Wilken et al., 2019), including data pertaining to how often and how many visibility boosts are bought by specific individuals, suggests that it is well within their ability to identify vulnerable users. For another, even if such identification were somehow impossible, there would still be *general measures* that these companies could take to protect people from overspending on visibility boosts. For example, they could introduce waiting periods before users can purchase their next boost; place limits on the amount of money that users can spend on such boosts; and refrain from sending notifications that encourage users to boost.

## The socio-economic objection

A second objection against the unfettered sale of visibility boosts maintains that this practice helps to create socio-economic injustices. In premise-form, it might be formulated thus:

1. Intimate relationships have a major positive influence on most people’s well-being.
2. When a given good has a major positive influence on most people’s well-being, justice requires that companies do not deny individuals fair access to it.

Therefore,

3. Justice requires that companies do not deny individuals fair access to intimate relationships.
4. When people can buy visibility boosts without any restrictions, many of those unable to afford such boosts will lack fair access to such relationships.

Therefore,

5. The unfettered sale of visibility boosts is unjust.

Since various studies (for an overview, see Ge et al., 2020) have shown that intimate relationships are crucial to most people’s wellbeing (premise 1), I will focus here on vindicating premises 2 and 4.

The best way to defend premise 2 (‘When a given good has a major positive influence on most people’s well-being, justice requires that companies do not deny individuals fair access to it’) is to point out that the principle that we ought to have fair access to goods that are essential for (almost) everyone’s wellbeing is already accepted widely, and I believe correctly, within various other contexts. For example, it is because minors and adolescents are not morally responsible for their parents’ level of wealth that it appears unfair, and therefore unjust, when some of them are able to receive a significantly better education than their peers *simply because* their parents have deeper pockets (cf. Brighouse & Swift, 2014).^4^ Likewise, it seems that it would have been unfair, and therefore unjust, had governments allocated Covid-19 vaccines during the recent pandemic based on people’s ability and willingness to pay for these vaccines (cf. Rhodes, 2021), which might be partially why most of them used alternative distributive criteria such as vulnerability instead. (Notice that even a libertarian such as Robert Nozick (2013, p. 179) accepts that there are certain goods, such as drinkable water, that may not in their entirety be appropriated or purchased by a single agent,^5^ but should instead be available on a more egalitarian or equitable basis even if the range of goods to which this applies is smaller for libertarians than it is for liberals such as John Rawls (1999) and Ronald Dworkin (1978) and does not necessarily include intimate relationships, as I assume here it should.)

To defend premise 4 (‘When people can buy visibility boosts without any restrictions, many of those unable to afford such boosts will lack fair access to intimate relationships), it bears mentioning that using visibility boosts does not just increase the boosters’ chances of gaining likes and matches and, ultimately, of finding romantic partners given the prominent role that dating apps have come to play in early-stage dating (see the previous section). It does so *at the expense* of the chances of non-boosting users to do so, as these individuals will necessarily become less visible on the relevant dating app-platforms (i.e. we are dealing with a zero-sum game). Now, the problem that arises is that when people use visibility boosts that other dating-app users cannot afford, this will often be made possible by an unfair division of resources. The easiest way of bringing this is out is to note that most dating app-users are individuals in their late teens and twenties,^6^ which is a stage of life during which differences in wealth seldom derive fully, or even largely, from merit, i.e. from people’s talents and their motivation to work hard.^7^ Instead, said disparities tend to be predominantly based on differences in parental wealth for which we are not morally responsible. However, if this is correct, then we are led to the conclusion that the unfettered sale of visibility boosts must generate socio-economic injustices by unfairly giving those from higher socio-economic backgrounds better access to likes and matches and, ultimately, to intimate relationships than those from lower socio-economic backgrounds.

A critic might reply that this argument proves too much. On this view, if the unfettered sale of visibility boosts is unjust by unfairly restraining the opportunities of individuals from lower socio-economic backgrounds to find love, then the *same must be true* of the sale of premium memberships on dating apps, which might be deemed a *reductio ad absurdum*. In support of this conclusion, our critic may adduce the following observations. The first is that, like visibility boosts, premium memberships raise people’s chances of acquiring (additional) likes and matches and, in so doing, their chances of establishing intimate relationships, which may be due to several factors. These include, but are not necessarily limited to, the fact that premium members might be able to view and/or like more profiles than regular members; the fact that they might be able to search for partners in a more fine-grained manner; and the fact that they might be the only users who are able to see who liked them without matching first (Beck, 2021). The second observation is that, just as there are individuals who cannot afford visibility boosts through no fault of their own, so there are individuals – who for the reasons discussed in the previous paragraph will include many young people – who through no fault of their own cannot afford premium memberships.

My rejoinder to this criticism is to bite the bullet. In the same way that the sale of visibility boosts can be unjust on socio-economic grounds, I suspect that the sale of premium memberships on dating apps can be. Yet, there are grounds for doubting whether this is true of many premium memberships that are currently being sold. To see this, notice that, as it stands, the amounts of money that dating app-users can spend on premium memberships tend to be *much more limited* than the amounts that they can spend on visibility boosts. For example, whereas the most expensive Tinder membership (Platinum) will cost a 32-year-old in The Netherlands 30 EUR per month as of early 2022, a 12 h-Super Boost on this app will set him back 98 EUR (see the penultimate section). Although already a substantial amount, were he to (super)boost more than once a month, said individual can easily spend hundreds, if not thousands, of Euros on visibility boosts every single month, or indeed every single week.

To be sure, I am not denying that even within an affluent and relatively egalitarian country such as The Netherlands, a 30 EUR monthly membership fee will be prohibitively expensive for some people, just as premium membership fees within other countries – which are typically adjusted to reflect average local salaries – will be prohibitively expensive for certain segments of society. The reason why I do not think this is a decisive objection to the sale of premium dating app-subscriptions is that many, if not most, offline ways of meeting would-be romantic partners are *at least as expensive.* When one goes to a pub or a discotheque, one will normally pay for drinks and, in the case of the latter, sometimes pay an admission fee as well. Similarly, those who wish to meet potential partners at festivals or concerts will ordinarily need to purchase tickets to get in, whereas those hoping to find love at private parties are often expected to bring beverages or snacks, which will also come at a cost. When we factor this in and further consider that, like any other type of business, dating app-companies have legitimate interests in making a profit, it becomes difficult to see how it could be unreasonable for companies such as Tinder to charge the kinds of premium membership fees that they are currently charging. (Of course, this could change in the future if, and when, these fees are raised dramatically; my point is simply is that it is far from obvious that they are currently excessive.)

## Concluding remarks

That concludes my critique of the unfettered sale of visibility boosts on dating apps. As I argued, there are two major problems with this practice, namely that it is likely to exploit the impaired autonomy of certain users and that it creates socio-economic injustices. While this does not necessarily mean that the sale of such boosts ought to be banned as happened with the sale of loot boxes within Belgium, which we saw are highly similar virtual items, it does suggest that, at the very least, states should regulate the market in them, which to the best of my knowledge no state is presently doing. As was mentioned, such regulations might include, but are not necessarily limited to, forcing dating app-companies to introduce waiting periods between purchases of visibility boosts; requiring them to cap the amount of money that users can spend on such boosts; and prohibiting them from sending notifications that encourage users to boost.

Let me end by noting that, while I have shown that visibility boosts satisfy a widely accepted definition of gambling and, design-wise, bear striking similarities to slot machines and loot boxes that have been found to be very addictive to some users, the question of whether they are indeed addictive and, if so, to what degree, will ultimately need to be settled by empirical research. My hope is that this contribution will stimulate scholars to conduct said research and that this will in turn enable philosophers and public policy-experts to make informed policy recommendations about potential bans and regulations for which I argued there is a *prima facie* strong case.

## Footnotes

1 Versions of this definition are also endorsed by e.g. Hodgins et al. (2011, p. 1874) and Potenza et al. (2001, p. 141), as well as by organisations such as the Australian Productivity Commission and US National Research Council (Williams et al., 2017, p. 9) and by online dictionaries such as those of Marriam Webster and the Encyclopaedia Britannica.

2 As they write, ‘while these features [Boost and Super Boost] don’t guarantee a match, they are incredibly effective in upping your chances, even after the Boost is over. Activate a Boost or Super Boost, and start swiping to see results’ (*Used Boost or Super Boost, and Didn’t Get Any New Matches*, n.d.).

3 As its Gambling Authority wrote, ‘prizes need not have monetary value’ or be convertible to something of such value for the Gambling Act to apply; what matters instead is that ‘players attach value to them [the items that can be won] and that this value is also stressed by the game developers’, for example by ‘giving colour or number-codes to express their value’ (Kansspel Comissie, 2018), which we have seen is something that Tinder does by marking purple the profiles of matches that were acquired while boosting.

4 This helps to explain why some countries, such as the Scandinavian ones, provide free higher education to their citizens and why even a more neoliberal country such as the United States has many universities and colleges that offer tuition-fee discounts to students from lower socio-economic backgrounds.

5 The reason for this offered by Nozick is that to do so would violate the Lockean proviso (which he endorses), according to which ‘a process normally giving rise to a permanent bequeathable property right in a previously unowned thing will not do so if the position of others no longer at liberty to use the thing is thereby worsened’ (Nozick, 2013, p. 178).

6 For example, polling by the Pew Research Center has found that 48% of 18- to 29-year-old Americans say that they have used a dating site or app as opposed to 38% of 30–39-year-olds; 19% of 50-65-year-olds; and 13% of those aged 65 and above (Vogels, 2020).

7 Which is not to suggest that this necessarily changes at later life stages. I remain non-committal on this here.

## References

[CR2] Alexopoulos C, Timmermans E, McNallie J (2020). Swiping more, committing less: Unraveling the links among dating app use, dating app success, and intention to commit infidelity. Computers in Human Behavior.

[CR3] Beck, R. (2021, April 24). *Dating apps: Is it worth paying a premium to find love?* The Guardian. http://www.theguardian.com/lifeandstyle/2021/apr/24/dating-apps-premium-find-love-over-30

[CR5] Blomley v Ryan (HCA 81 1956).

[CR6] Boost (2021). (n.d.). Tinder. Retrieved September 9, from https://www.help.tinder.com/hc/en-us/articles/115004506186-Boost

[CR7] Brady A, Prentice G (2021). Are loot boxes addictive? Analyzing participant’s physiological arousal while opening a loot box. Games and Culture: A Journal of Interactive Media.

[CR8] Brighouse, H., & Swift, A. (2014). *Family values: The Ethics of parent-child Relationships*. Princeton University Press.

[CR10] Brooks, M. (2019, January 4). *The “Vegas Effect” of Our Screens* | *Psychology Today*. https://www.psychologytoday.com/us/blog/tech-happy-life/201901/the-vegas-effect-our-screens

[CR9] Brooks GA, Clark L (2019). Associations between loot box use, problematic gaming and gambling, and gambling-related cognitions. Addictive Behaviors.

[CR11] Close, J., & Lloyd, J. (2021). *Lifting the Lid on Loot-Boxes*. https://www.begambleaware.org/sites/default/files/2021-03/Gaming_and_Gambling_Report_Final.pdf

[CR12] Coduto KD, Lee-Won RJ, Baek YM (2020). Swiping for trouble: Problematic dating application use among psychosocially distraught individuals and the paths to negative outcomes. Journal of Social and Personal Relationships.

[CR14] Colburn, B. (2010). *Autonomy and liberalism*. Routledge.

[CR15] Commercial Bank of Australia Ltd v Amadio (HCA 14 1983).

[CR16] Curry, D. (2021, August 24). *Dating App Revenue and Usage Statistics (2021)*. Business of Apps. https://www.businessofapps.com/data/dating-app-market/

[CR18] Dowling N, Smith D, Thomas T (2005). Electronic gaming machines: Are they the “crack-cocaine” of gambling?. Addiction (Abingdon England).

[CR19] Drummond A, Sauer JD (2018). Video game loot boxes are psychologically akin to gambling. Nature Human Behaviour.

[CR20] Drummond, A., Sauer, J. D., Hall, L. C., Zendle, D., & Loudon, M. R. (2020). Why loot boxes could be regulated as gambling. *Nature Human Behaviour*, *4*(10), 10.1038/s41562-020-0900-3. 10.1038/s41562-020-0900-3 32601458

[CR21] Dworkin, R. (1978). *Taking Rights Seriously* (Fifth Printing edition). Harvard University Press.

[CR22] Ge, F., Lembke, J., & Pietromonaco, P. R. (2020). Intimate Relationships and Physical Health. *The Wiley Encyclopedia of Health psychology* (pp. 337–345). John Wiley & Sons, Ltd. 10.1002/9781119057840.ch83.

[CR23] Griffiths M, Parke J, Wood R, Rigbye J (2010). Online Poker Gambling in University students: Further findings from an online survey. International Journal of Mental Health and Addiction.

[CR24] *[Guys] How many matches do you get from a boost?* (2018, December 11). [Reddit Post]. R/Tinder. www.reddit.com/r/Tinder/comments/a56tzb/guys_how_many_matches_do_you_get_from_a_boost/

[CR25] Harris A, Gous G, de Wet B, Griffiths MD (2021). The relationship between gambling event frequency, Motor Response Inhibition, Arousal, and dissociative experience. Journal of Gambling Studies.

[CR26] Henden E, Melberg HO, Rogeberg O (2013). Addiction: Choice or compulsion?. Frontiers in Psychiatry.

[CR27] Hodgins DC, Stea JN, Grant JE (2011). Gambling disorders. The Lancet.

[CR28] Hogarth L (2020). Addiction is driven by excessive goal-directed drug choice under negative affect: Translational critique of habit and compulsion theory. Neuropsychopharmacology : Official Publication Of The American College Of Neuropsychopharmacology.

[CR29] Kakavas v Crown Melbourne Ltd (HCA 25 2013).

[CR30] Kansspel Comissie (2018). *Onderzoeksrapport Loot boxen*. Kansspel Comissie. https://www.gamingcommission.be/opencms/export/sites/default/jhksweb_nl/documents/onderzoeksrapport-loot-boxen-final-publicatie.pdf

[CR31] Kent, C. (2015, January 20). *Tinder review: A woman’s perspective*. The Telegraph. https://www.telegraph.co.uk/men/relationships/10317832/Tinder-review-a-womans-perspective.html

[CR33] Linnet J, Rømer Thomsen K, Møller A, Callesen MB (2010). Event frequency, excitement and desire to gamble, among pathological gamblers. International Gambling Studies.

[CR34] Maslow AH (1943). A theory of human motivation. Psychological Review.

[CR35] Meisenzahl, M. (2020, August 6). *These charts from Match Group show more people are turning to online dating during the pandemic*. Business Insider Nederland. https://www.businessinsider.nl/tinder-hinge-match-group-dating-apps-more-users-coronavirus-2020-8/

[CR37] Nozick, R. (2013). *Anarchy, State, and Utopia* (2 edition). Basic Books.

[CR38] Potenza MN, Kosten TR, Rounsaville BJ (2001). Pathological Gambling. JAMA.

[CR40] Rawls, J. (1999). *A Theory of Justice* (Revised edition). Belknap Press.

[CR41] Reuters (2021, February 2). Match tops sales estimates as Tinder, Hinge keep sparks flying. *Reuters*. https://www.reuters.com/article/us-match-group-results-idUSKBN2A22V1

[CR42] Rhodes R (2021). Justice in COVID-19 vaccine prioritisation: Rethinking the approach. Journal of Medical Ethics.

[CR43] Roy, E. A., & Jong, E. (2017, August 7). Ban alcohol from supermarkets, urges New Zealand medical authority. *The Guardian*. https://www.theguardian.com/world/2017/aug/07/ban-alcohol-from-supermarkets-urges-new-zealand-medical-authority

[CR44] Schüll, N. D. (2012). Addiction by design: Machine gambling in Las Vegas. *Addiction by design*. Princeton University Press. 10.1515/9781400834655.

[CR46] Statista (2019). *Netherlands: Dating app use 2019*. Statista. https://www.statista.com/statistics/808067/most-popular-dating-apps-in-the-netherlands/

[CR47] *Super Boost*. (n.d.). Tinder. Retrieved September 9, from https://www.help.tinder.com/hc/en-us/articles/360029087891-Super-Boost

[CR48] Thal SN (1988). The inequality of bargaing power doctrine: The problem of defining contractual unfairness. Oxford Journal of Legal Studies.

[CR49] Timmermans E, De Caluwé E (2017). Development and validation of the Tinder Motives Scale (TMS). Computers in Human Behavior.

[CR50] Turner, N., & Horbay, R. (2004). How do slot machines and other electronic gaming machines actually work. *J Gambl Issues*, *11*. 10.4309/jgi.2004.11.21

[CR52] *Used Boost or Super Boost, and didn’t get any new matches*. (n.d.). Tinder. Retrieved September 11, from https://www.help.tinder.com/hc/en-us/articles/115003638126-Used-Boost-or-Super-Boost-and-didn-t-get-any-new-matches

[CR53] Vandaele Y, Ahmed SH (2021). Habit, choice, and addiction. Neuropsychopharmacology : Official Publication Of The American College Of Neuropsychopharmacology.

[CR54] Vega, N. (2019, January 26). Tinder settles age discrimination lawsuit for $17.3 million. *New York Post*. https://nypost.com/2019/01/25/tinder-settles-age-discrimination-lawsuit-for-17-3-million/

[CR55] Vogels, E. (2020, February 6). 10 facts about Americans and online dating. *Pew Research Center*. https://www.pewresearch.org/fact-tank/2020/02/06/10-facts-about-americans-and-online-dating/

[CR56] Volkow ND, Koob GF, McLellan AT (2016). Neurobiologic advances from the Brain Disease Model of Addiction. New England Journal of Medicine.

[CR57] Wiers RW, Verschure P (2021). Curing the broken brain model of addiction: Neurorehabilitation from a systems perspective. Addictive Behaviors.

[CR58] Wilken, R., Burgess, J., & Albury, K. (2019). Dating Apps and Data Markets: A Political Economy of Communication Approach. *Computational Culture*, *7*. http://computationalculture.net/dating-apps-and-data-markets-a-political-economy-of-communication-approach/

[CR59] Williams, R. J., Volberg, R. A., Stevens, R. M., Williams, L. A., & Arthur, J. N. (2017). *The definition, dimensionalization, and assessment of gambling participation*. Canadian Consortium for Gambling Research.

[CR61] Winters, K. C., Chung, T., Stinchfield, R., Kassel, J. D., & Conrad, M. (2012). Addictions and Adolescence. In V. S. Ramachandran (Ed.), *Encyclopedia of Human Behavior (Second Edition)* (pp. 9–21). Academic Press. 10.1016/B978-0-12-375000-6.00004-5

[CR62] Zendle D, Cairns P (2018). Video game loot boxes are linked to problem gambling: Results of a large-scale survey. PLOS ONE.

[CR63] Zendle D, Cairns P (2019). Loot boxes are again linked to problem gambling: Results of a replication study. PLOS ONE.

